# Navigating unique challenges: librarian perceptions in supporting physician associate (assistant) programs

**DOI:** 10.5195/jmla.2026.2211

**Published:** 2026-01-01

**Authors:** Megan Jaskowiak, Michelle Nielsen Ott, Karina Kletscher

**Affiliations:** 1 jaskowma@miamioh.edu, University Libraries, Miami University, Oxford, OH; 2 mnielsenott@bradley.edu, Bradley University, Cullom-Davis Library, Peoria, IL; 3 karinakletscher@creighton.edu, Creighton University, Health Sciences Campus, Phoenix, AZ

**Keywords:** Physician assistant (associate) education, health sciences librarianship, resource management, library instruction, librarian workload

## Abstract

**Objectives:**

This study examines the experiences of librarians who support physician assistant/associate (PA) programs, describing the unique challenges of these programs and outlining strategies that librarians adopt to engage these programs.

**Method:**

This mixed-methods study includes two phases: (1) a quantitative survey developed and distributed to library personnel in institutions with established or developing PA programs in the US and Canada, and (2) semi-structured interviews with fifteen selected survey respondents, focusing on their experiences and perceptions related to PA education support. The qualitative data were analyzed using thematic analysis.

**Results:**

Seventy-five survey responses were collected. Key findings from the survey include: most respondents were from universities with health sciences programs, with nursing and physical therapy being the most common additional programs. Most library-led instruction occurred during the didactic phase and focused on search skills and evidence-based practice. PubMed and UpToDate were the most library-promoted resources. Two thematic elements discovered through the semi-structured interviews were “relationship building as paramount” and “impact of the learning curve on librarian workload.”

**Conclusion:**

Librarians who support PA educational programs face challenges related to relationship building, financial resources, workload, and steep learning curves. The findings underscore the need for targeted professional development programs to equip librarians with the necessary knowledge and skills.

## INTRODUCTION

Physician assistant or associate (PA) programs have rapidly expanded in response to the growing demand for advanced care providers in the United States [1]. According to the Bureau of Labor Statistics, this profession will grow 28% between 2023 and 2033 [2]. The growth of PA programs has outpaced many other healthcare professions, highlighting the need for comprehensive educational support systems for these clinicians [1,3]. In 2024 alone, 20 new PA programs were under development, adding to the 217 accredited programs already graduating thousands of healthcare professionals annually [4]. The emerging needs of these PA programs have created challenges for their parent institutions, including academic and health sciences libraries.

PA students may seem simultaneously similar and different from their other health professions counterparts. Like these peers, PA students complete a rigorous postbaccalaureate two-phase program consisting of didactic coursework followed by clinical rotations [5]. This education is on a compressed timeline, with some PAs earning their degree in as little as 12 months, notably shorter compared to the traditional four-year medical degree. Similar to doctors, PAs are educated as generalists in the medical model, with the exception of a second year of more specialized clinical rotations [5]. PAs may practice upon graduating and certification without additional training, such as fellowships or residencies. PAs may be seen as more akin to nurse practitioners (NPs) in terms of clinical settings and roles, but NPs are educated in the nursing model and must also complete advanced education and clinical training beyond their initial registered nurse (RN) education [6,7]. Outside of a bachelor's degree and prerequisites, PAs may come from any former career path or academic discipline. Additionally, as generalists, PAs do not earn certification in a particular population of focus as their NP colleagues, though PAs may certainly go on to gain additional education and practice in a clinical specialty [8,9].

In 2018, 222 libraries were identified as supporting PA programs [10]. Despite the increasing number of programs, there is a significant gap in the literature regarding the role of librarians in PA education. Since the PA curriculum spans multiple specialties and topic areas, librarians rely on each other because core authoritative resources and faculty information-sharing are inconsistent or wholly unavailable. [11]. Thanks to the work of library peers, the PA librarian can now refer to resources on PAs and evidence-based medicine (EBM) as well as full bibliographies to support collection development, resource management, and general reference [10–16]. Resource evaluations report that larger institutions, particularly those with existing medical programs, provided more materials and subscriptions applicable to their PA programs, indicating that a librarian building a collection from the ground up requires adaptability in negotiating costs and balancing freely available resources with subscriptions, if not a significant budget [10,12–16].

While the Medical Library Association (MLA) provides a framework of core competencies for health sciences librarianship, these guidelines cannot fully account for the distinct pedagogical, clinical, and accreditation contexts that shape PA education [17]. As a result, librarians serving PA programs often lack a clear avenue to explore how their professional skills translate into this specialized environment. Foreman and Baldwin captured librarians’ experiences and perceptions of liaising to the relatively new profession in 1976 [18]. As both the PA and library professions have significantly evolved over the last 50 years, revisiting the role of PA librarians is essential to highlight the unique challenges and contributions of this role, foster a more informed and supportive professional community, and guide the development of targeted resources and training. In an effort to address this gap, this study explored librarians' perceptions of their own work and experiences with PA programs.

## METHODOLOGY

This research project included two phases. In the first phase, a 10-question survey was developed in Qualtrics (Appendix A). The survey design was informed by the researchers’ knowledge of PA programs and previous research on librarians working with health sciences programs. The survey was designed to collect baseline information about libraries and librarians supporting PA programs and to recruit participants for the semi-structured interviews in the second phase of the project. The survey was reviewed by librarians who were not part of the study before deployment. In March 2023, upon IRB approval from the Miami University Human Subjects Committee (Protocol ID# 04470e), an email invitation to participate in the survey was sent to the MLA mailing list and directly to 277 librarians listed on library websites at institutions with PA programs. The survey required respondents to be at least 18 years old and employed as a librarian or information specialist at an institution with either an established PA program or in the process of implementing a PA program in the United States or Canada. Descriptive statistical analysis was performed on the survey responses using Excel. The survey's final question asked the respondents about their willingness to participate in an interview.

The second phase consisted of semi-structured interviews. The 36 survey respondents who indicated willingness to be interviewed were categorized based on their institution type and the length of time since program accreditation. The interviewees were selected randomly from within these designations (Appendix B) to ensure representation in the two categories and institutions across the United States and Canada. Three of the initially selected interviewees did not respond to the interview request; three different interviewees were chosen from the original pool. The researchers were each assigned five people to interview based on their respective time zones for a total of 15 interviews, corresponding to standards of saturation [19]. Using a set of semi-structured interview questions devised by the research team, 15 semi-structured interviews were conducted between July and December 2023 (Appendix C).

The semi-structured interviews were conducted and recorded over Zoom. Interviewees had the option to have their cameras on or off. The Zoom-generated transcripts from these sessions were reviewed and edited by the interviewing researcher to ensure accuracy. Once all transcripts were edited and finalized, each researcher was randomly assigned five transcripts to begin the thematic analysis technique described by Braun and Clarke [20]. Taguette, an open-source qualitative research tool, was used for this analysis. As a web-server-based tool, Taguette provided a collaboration space for coding among the researchers [21]. To start the analysis, each researcher created concept keywords by reading the transcripts and noting common sentiments expressed by the interviewees. Then the researchers met and discussed the keywords and combined the concepts to create a set of data-derived codes with agreed-upon definitions. These codes were applied to the previously randomly assigned transcripts using Taguette. The researchers then identified patterns and developed, revised, and defined themes.

## RESULTS

### Quantitative Survey Results

A total of 87 quantitative survey responses were collected, with 75 surveys containing at least one response to one of the questions. Twelve respondents opened the survey but answered no questions, while one respondent skipped multiple questions. Assuming one response per institution, this represents approximately 25% of institutions with accredited PA programs (219 fully accredited and 81 provisionally accredited). Although the survey sampling was self-selected, the respondents represented similar percentages in the categories of length of time since accreditation as the ACR-PA data at the time of survey data collection.

Most respondents were employed at a college or university with a dedicated health sciences/medical school or one with graduate programs. The rest of the respondents were from very diverse types of institutions, including hospitals, a liberal arts college, graduate health science schools, a community college, and an osteopathic school.

All respondents reported that their institutions supported additional health sciences programs either at the undergraduate or graduate level (Table 1). The most common other health science program supported was nursing, including undergraduate or graduate programs at 79%, followed by occupational/physical therapy programs with 75%. Overall, respondents indicated that other allied health programs were more common to have at their institution than having a medical school at their institution.

**Table 1:** Other Health Sciences/Medical Programs at the institutions (both absolute total respondents and percentages). The percentage totals will not equal 100% due to the nature of a multiple-response question. N=75

Other Health Sciences/Medical Programs	Totals (%)
Nursing	56 (79%)
PT/OT	53 (75%)
Public Health	45 (63%)
Biology/Biomedical Sciences	42 (59%)
Exercise Physiology/Athletics/Kinesiology	41 (57%)
Medicine	40 (56%)
Pharmacy	30 (42%)
Dentistry	21 (30%)
Osteopathy	12 (17%)

As for library-led instruction, respondents reported that these sessions most frequently occurred during the didactic phase of the PA program. The didactic phase of a PA program varies by institution and program. Sixty-five percent of the respondents indicated instruction occurring only in didactic classes, 18% indicated library-led instruction in both didactic and clinical rotations, and another 16% indicated either no instruction or not associated with a particular class (Table 2). For the institutions with library-led instruction, teaching general search skills and developing search strategies were the most frequent topics (83%). Respondents who indicated that they taught in didactic and clinical rotation classes were more likely to cover EBM topics in the instruction sessions.

**Table 2:** Library instruction responses were divided and categorized by location and type of instruction. Type of instruction totals will not equal 100% due to the nature of a multiple-response question. A single respondent indicated that they only did instruction in the clinical setting. This information has been incorporated into the percentage calculations but is not shown in the table. N=74

Types of classes	General search skills	Clinical health sciences tools	Developing search strategies	Evidence-based practice
**Didactic: 48 (65%)**	41 (85%)	35 (73%)	41 (85%)	29 (60%)
**Both: 13 (18%)**	12 (92%)	12 (92%)	13 (100%)	12 (92%)
**Not specifically associated with a class: 12 (16%)**	10 (83%)	10 (83)	8 (67%)	7 (58%)
**Total:**	63 (85%)	57 (77%)	62 (84%)	48 (65%)

When asked which library products/library resources are promoted to the PA programs, PubMed (94%) was the most common overall, as well as the most common article database. CINAHL was promoted by only 59.4% of the respondents. Seventy-two percent of the respondents indicated that they promoted AccessMedicine. As for clinical care tools, more indicated they had UptoDate (65%) compared to either Dynamed (27%) or Clinical Key (34%). Nine respondents indicated that they had Dynamed but neither UpToDate nor Clinical Key. Three institutions had both Dynamed and Clinical Key, and one institution had all three clinical care tools. VisualDx, Lexicomp, and StatRef were the least promoted products.

Sixty-eight percent of the respondents indicated that the PA program at their institution includes a research component, while the rest responded that they were unsure or that there was no research component. Those who gave affirmative answers were prompted to provide open-ended answers about the research component and the library's participation (if any) with the students for the research component. Thirteen reported that students were required to complete a capstone project involving research or a review (such as literature, narrative, or 'mini' systematic review). Seven reported that librarians had minimal involvement in the research project, while 13 provided specific instruction sessions during the second year when students were actively conducting their research. Additionally, six respondents indicated that they offered consultations or workshops. Finally, nine respondents mentioned that they either led the course or were embedded in the course, where students conducted their research.

### Qualitative Semi-Structured Interview Results

The thematic analysis of 15 semi-structured interviews revealed two main themes, each with two subthemes (Table 3 and Table 4). The first main theme, “relationship building as paramount,” is supported by the subthemes “proximity has value” and “external perception of librarian/library affects the role of the librarian/library.” The second main theme is “impact of the learning curve on librarian workload” accompanied by the subthemes, “PA programs/students as unique” and “financial barriers while trying to meet resource needs of PA programs.” These overarching themes emerged across each of the interviews conducted, regardless of whether the participant: worked at a nascent or a well-established PA program; had limited or lengthy professional experience; or had a limited or robust collections’ budget.

**Table 3:** Subthemes and exemplar quotes for Theme 1, “Relationship building is paramount.” “Relationship building is paramount” describes how librarians that establish a working relationship with the PA programs experience more success broadly.

Subtheme	Exemplar Quotes
**Proximity Has Value:** building relationships with the PA programs feels easier when the library is co-located with PA students and faculty.	Proximity to students “[The library] is where the PA students live pretty much their entire students live pretty much their entire didactic year.” Proximity to faculty “…we just have so much interaction with them because we’re constantly going up and down that elevator and I’m just catching, I’m just I’m you know every chance I get I’m gonna share with them.”
**External Perception of Librarian/Library Affects the Role of the Librarian/Library:** a PA program's prior held perceptions or beliefs affects a librarian's success at building relationships	Not valued or undervalued “The faculty would, quite honestly, to my face, tell me that they don’t really use library resources… and they say the students have to find scholarly, peer-reviewed articles related to medicine [but] don’t know [how] they find them.” “They may already have a sort of perception about what the library does and then maybe that's so because of that and maybe the only reason they reach out to me is for what they expect the library does.” Valued “My faculty colleagues gained a confidence and trust in my abilities. They’ve been really fantastic to collaborate with.” “...any help that you can do with them in the accreditation process…I find that really builds a lot of goodwill. So. I think, I don't know, I just, it's a lot of work, but I think it's very rewarding”

**Table 4:** Subthemes and exemplar quotes for Theme 2, “Impact of the learning curve effects on librarian workload.” This theme describes how PA programs are distinct from other health sciences programs and how developing an understanding of their unique needs takes time and educational resources.

Subtheme	Exemplar Quotes
**PA Programs and Students are Unique:** PA students come from different bachelor's degree programs than other health sciences fields. Their compact schedule means they are often occupied during a librarian's traditional working hours.	Distinct among health sciences “And I don't think they liked being sort of lumped in with nurses, they didn't like being called 'Doctors-lite,' and it was more stuff that was started specifically for them…” “Because in PA they kind of cover everything. But they also have a unique identity and occupy this weird space.” Scheduling conflicts “So the vast majority of times, I was helping students via email because they wouldn't be able to talk to me until 8 pm.” Diverse students’ backgrounds “It is really focused on the medicine and it's interesting so I would say like a lot of the PA students, their background is very different and they all come from very different backgrounds. And I've seen more and more people coming from nursing and from OT or PT backgrounds.”
**Financial Barriers for Meeting the Resource Needs of PA Programs:** providing library resources to support PA programs often requires working within financial constraints.	No financial barriers “I feel like we because we have the medical program and really a lot of the resources that they use, the med students do too. So, in terms of [things] like funding and that sort of thing, that's fine.” Funding issues “So like UpToDate, clinical consult tools, UpToDate, AccessMedicine, anatomical guides, these sort of things…they are owning and managing their own subscriptions or products for those” “…our new health sciences programs, they're not budgeted the same way as the rest of the university… They're coming out of special investment strategic funds…[they] have their own library budget, so the library does not pay for their resources unless we already had the resources.” “We have cut things that are needed because our budgets can't absorb the inflation costs” “…Dynamed, which is less expensive…so we switched to that”

### Theme 1.0 Relationship Building as Paramount

Building relationships between a library and a PA program can be fraught due to librarians and teaching faculty having different responsibilities and priorities. Librarians are often brokering acquisition and access as well as navigating requests from library users and administrators, or what one participant called “the business side of being a librarian.” Interviewees described upholding relationships with PA faculty and students built in the classroom while maintaining library resources and services as a tricky balancing act of “trying to keep both sides happy.” Another participant recounted an experience with a PA program director who was “wanting these things, and I'm like, at the time, I was told no because we didn't have the money…it got all sorts of uncomfortableness…we're just going to have to see what happens.”

Interviewees' relationships with their PA programs varied. Several interviewees reported that they were able to slip easily into positive collaborations inherited from previous liaisons. In contrast, due to the rapid growth of PA programs, new and untested relationships often arose between the library and the emerging program when attempting to sort through accreditation requirements. Some interviewees established positive, professional relationships with their PA programs through accreditation (both provisional and continued statuses) and instruction.

Interviewees indicated that leading library instructional programs was central to their relationship with the PA program. Library instruction opportunities varied in both delivery modes and course content, from multi-hour orientations to 60-minute one-shots to integrated scaffolded sessions. One participant shared their experience as a co-faculty in a PA research methods course, but they warned, “It's probably hard to talk your way into it [instruction]” without research-centric coursework or with faculty who are “skeptical about what I [the librarian] could do for them.”

Interviewees discussed the pivotal figure of a library champion who refers colleagues and students, invites the librarian into classroom instruction, and collaborates in collection and resource development. They indicated the value and variety of library champions, including individual faculty, the program director, staff (e.g., the clinical coordinator or administrative assistant), and students. Interviewees expressed that library champions with word-of-mouth advertising catalyzed multiple collaboration opportunities. One participant shared how this phenomenon has become their general approach to relationship-building: “I almost feel like it's that snowball effect, like you get one or two people who are excited about how you supported them. They'll talk to their colleagues about how a librarian supported them in the classroom.”

Another common experience, interviewees shared is the continued hope and perseverance to expand and increase relationships and opportunities with their PA programs. “I’m hopeful you know it's kind of a long game.” As a different participant notes, “developing those relationships takes time and effort,” a luxury not all librarians have. Still, participants shared that the investment pays off.

### Subtheme 1.1 Proximity Has Value

Interviewees reported that the locations of the library and the PA program affected relationship building and student use of the library. Interviewees observed PA students’ steady usage of physical library spaces when the library was conveniently located to the program (i.e., classes or residential housing). They also shared that physical library space – “outside of their normal classroom” – is valuable for PA students for quiet study or facilitating group work, particularly during evening hours when students are done with didactic or clinical work. As one stated, “[the library] is where the PA students…live pretty much their entire didactic year.”

Interviewees working at libraries located further from the core class activity reported distance as a barrier. They observed how geographic hurdles, such as programs based in disparate locations or students who don’t live in student housing, do not use the library's physical space; as one participant describes students in the PA program as “not that they're isolated, but they're in their own space.” Another states, “[it] would be nice to change if they were physically closer, and so it was more convenient for me to be there and for them to be in the library.” Similarly, interviewees felt that physical proximity to PA faculty and staff created more opportunities for personal connection and serendipitous liaising. For example, “...we just have so much interaction…going up and down that elevator…every chance I get I’m gonna share with them.” Consistent facetime with program constituents can mitigate obstacles, concerns, and gaps, such as “what's working, what's not working, what changes might need to be made,” as one participant listed, and is most helpful to understanding library resources.

### Subtheme 1.2 External Perception of Librarian/Library Affects the Role of the Librarian/Library

The interviewees’ perceptions of how the faculty/staff in their PA programs perceived their role in the program varied greatly. At one end of the spectrum were those who felt they were not valued at all, as one said, “the biggest detriment is the administration. They don't value the library or don't understand the value of the library…as a whole, there might be few people that do, but they don't have a loud enough voice.” Another stated, “the faculty would, quite honestly to my face, tell me that they don't really use library resources and they say the students have to find scholarly, peer-reviewed articles related to medicine [but] don’t know [how] they find them.” Conversely, some felt like they were perceived as colleagues who could play a pivotal role in the program with one interviewee stating, “The library is seen as a key player…[and] I'm very well received over at the PA program.” Interviewees noted issues regarding preconceived notions about library/librarian roles. One reported, “they may already have a sort of perception about what the library does…because of that, and maybe the only reason they reach out to me, is for what they expect the library does.”

### Theme 2.0: Impact of the Learning Curve on Librarian Workload

Interviewees discussed the added work and cognitive load associated with liaising with PA programs. As more PA programs are added to institutions across the United States and Canada, the work of providing library services and resources is added to the portfolio of health science librarians. Multiple interviewees reported fewer librarian positions at their institution but more programs and students to support. One interviewee expressed, “I find it's more demanding than the other health sciences programs…maybe it's because it's a new program.” A learning curve was expressed by interviewees about starting a new health science librarian position that supports PA programs. As one recalled, “I didn't even know what a PA was, basically, until I took the job.”

Additionally, demonstrating competencies added to the workload of the interviewees who reported needing to prove their skills. One interviewee stated, “I think there was a healthy level of skepticism when I first started. But as I've demonstrated my skills and what a librarian can bring to the program and how we can support, they've been much more receptive.”

Interviewees recounted that balancing the responsibilities from the business side and teaching sides of librarianship added a complicated layer to the librarian workload, status, and recognition. They commonly shared the difficulty of reaching and connecting with PA students inside and outside of the classroom. One said, “There's no bandwidth for anything extra right now. So it's interesting for me to figure out how to navigate so that I can provide the support that the program needs.” One participant estimates that it “…is individual and group instruction that takes up probably 60% of my time.” Another stated, “I need to figure out how to balance things.” The need for more institutional support was also apparent, as one said, “I think it would be really wonderful if libraries also considered what kind of support the librarians need.”

### Subtheme 2.1 PA Programs/Students as Unique

PA programs are unique compared to other health sciences programs. Interviewees from institutions with medical schools reported that the PA program aligns closely with the medical school. However, for PA programs at institutions without a medical school, interviewees experienced PA programs and PA students inhabiting a space between medical and allied health programs.

Interviewees reported that their experiences with the compact curriculum of the PA program created a barrier for librarians to interact with PA students and faculty outside of the classroom. Due to the packed structure of the program, PA students and faculty spend much of the traditional working hours of a librarian (9 am to 5 pm) in class or on clinical rotations. This schedule makes matching availability for meetings and consultations difficult, especially in the clinical phase. Interviewees expressed the need to offer virtual appointments and instruction to accommodate busy schedules (including during evenings and weekends).

As discussed in the previous themes, the frequency with which librarians interacted with PA students through instruction or research depended on the institution. Some interviewees reported less contact with the PA faculty and students compared to other programs they work with. There is “not as much [contact] in comparison to nursing, for example”. However, this was not the case for all interviewees. Some reported that PA students regularly use library resources and schedule consultations with librarians for research assistance, but the majority of these interactions occurred at specific times, for example, during orientation, in the research methods course, or for a capstone project.

Another unique aspect interviewees described about their interactions with PA students is the students’ diverse educational and experiential backgrounds. Librarians usually experienced graduate students in allied health programs and medical schools with an educational background in their field from their undergraduate studies; this was not the case with PA students. The diverse backgrounds of students created a challenge for librarians to meet the instructional needs of the students. As one interviewee put it, “...the thing I find the most challenging with them is because they're mature students and they're coming from all these different backgrounds, trying to teach them at the start of the program. It's, you don't really know where they're at."

### Subtheme 2.2 Financial Barriers While Trying to Meet Resource Needs of PA Programs

Not having the appropriate budget for the library resources needed (or wanted) by the PA program was mentioned frequently in interviews. Almost all interviewees spoke of some type of budgetary issue or financial support issue for access to resources. The few interviewees who did not express this issue were at institutions with a medical or osteopathic school, who spoke of financial issues that were ultimately related to how their budget was structured, instead of an affordability problem.

Of those interviewees who spoke about resource funding or budgetary issues, many described unique funding models. For example, one interviewee explained, “...we have an eclectic mix that's grown organically over the years as far as funding goes.” At a number of these institutions, while the library budget paid for most resources, departmental funds were used to pay for specific items/resources but were managed by the libraries. One interviewee stated, “We're not [financially] associated with the main campus library at all [which] creates…a huge barrier.” Other interviewees noted that the PA program paid for and administered the resources. Some worked at institutions where additional resources “are paid through student fees,” and others were at institutions that used strategic investment funds for new health sciences programs or money from state or federal programs. These unique financial situations created extra worries expressed by multiple interviewees. Some interviewees had to cut access or choose between resources, like switching from UpToDate to Dynamed.

## DISCUSSION

As PA programs have expanded rapidly, librarians have had to assume a greater responsibility for supporting these programs. Needing to build and maintain relationships, resource management challenges, and workload issues characterize the challenges of the PA librarianship.

Relationship building is an important aspect of all health sciences liaison positions [15]. This study finds that strong relationships with PA faculty enhance PA student education. PA librarians need to maintain a balance between business responsibilities and instructional responsibilities. Diverse instructional approaches, from multi-hour orientations to embedded co-teaching in research methods courses, demonstrate the need for flexibility in the challenge of supporting these programs. Interviewees who inherited positive collaborations from previous liaisons showed that established relationships can create lasting frameworks. For some, the physical proximity of librarians was a significant factor as library spaces located near PA programs were reported to be used more frequently, and faculty interactions were higher. In their study on faculty's perception of academic librarians, Weng and Murray also found that physical proximity had a positive effect on the faculty's perception of librarians [22].

PA programs occupy a unique niche in health sciences education. It's apparent from the interviewees that the medical education model influences every part of the experience for PA students, faculty, and librarians. However, they have unique needs even compared to medical schools or other allied health programs and understanding them is vital to effective librarianship. Librarians must contend with the intensive compressed curricula, creating scheduling and resource challenges as well as the diverse educational backgrounds of PA students, which complicates instructional design.

Most survey respondents reported library instruction only during the didactic portion, meaning PA students may not receive adequate support during their clinical phase. Some interviewees expressed interest in expanding their instructional reach, while others spoke of their satisfaction with the successful expansion, such as co-teaching in EBM classes. While the majority of respondents reported teaching general search skills, the opportunity to cover EBM topics was more common with respondents who taught in both didactic and clinical coursework. Involvement during the students' research component, which is typically toward the end of the program, varied significantly, ranging from minimal participation to leading instruction sessions, offering consultations or workshops, and full integration into research courses. While no study has examined the effects of multiple library instruction sessions across the PA curriculum, studies on other graduate-level medical and health science programs have concluded that the information-seeking skills benefited from this multi-level approach [23,24].

The reported financial barriers reflect broader trends in academic libraries. This situation is particularly acute at institutions without medical schools, where PA programs may represent the only program requiring high-cost resources, in particular, point-of-care tools. Studies have determined that institutions with a medical school have access to more resources [10,15]. With 28% projected growth in the profession [2] and 20 new PA programs under development in 2024 alone [4], more libraries will face increasing pressure to acquire specialized resources with limited budgets. The interviews also demonstrate the lack of standardization in supporting these programs. A core list for collection development has not been attempted since 2001 [16]. Johnson and Johnson attempted to fill this collection development gap by studying the LibGuides created by librarians for PA programs, concluding that they could be used to develop collections suitable for PAs [11]. However, Petersen [10] felt that Johnson and Johnson's list may be too limiting because it depended on programs that license Springshare software.

The “learning curve” described by many interviewees underscores a significant need for professional development opportunities to develop specialized knowledge. Many health sciences librarians do not have formal science or health science educational backgrounds [25]. This knowledge gap can create an additional workload for the librarians as they must pursue various avenues to gain the knowledge needed to understand these programs.

Library services are unevenly integrated within PA programs, ranging from librarians who feel unappreciated to those who are considered essential collaborators. Many factors contributing to this variability include the age of the program, the presence of library champions, involvement in accreditation processes, the individual librarian's approach to relationship building, and teaching faculty and student perceptions of librarians. Some interviewees suggest that librarians who actively participate in accreditation processes or who identify faculty advocates can significantly improve their integration within PA programs.

## IMPLICATIONS FOR PRACTICE

Providing library support to PA programs presents challenges for librarians because it requires specialized knowledge, flexibility in service delivery, and strategic relationships. It is essential to understand the unique characteristics of PA education, develop appropriate professional expertise, and position library services as essential to program success.

PA librarians have found themselves needing to quickly acclimate to a curriculum that, while rooted in the medical model, often includes students without clinical backgrounds or faculty without research backgrounds. This has required a shift in communication strategies, particularly moving away from assumptions about prior knowledge and toward more inclusive, plain-language approaches. Many librarians described immersing themselves in academic catalogs, board exam structures, and online PA student forums to better understand the pedagogical and cultural context of PA education.

The demands of PA program support have prompted librarians to rethink and expand beyond traditional liaison models and practices. Librarians have pivoted toward more proactive and embedded approaches, initiating contact with program directors early in the program's development and maintaining visibility through faculty meetings, curriculum planning, and informal social gatherings from water cooler chat to mixers. These efforts reflect a shift from transactional service delivery to sustained, peer-like engagement often requiring librarians to move outside the library's spaces.

Similarly, physical and scheduling constraints have prompted librarians to rethink how and where they offer support. In response to PA students’ limited presence on campus and/or in the library, librarians have shifted consultation hours, opted to travel to satellite locations, and leveraged asynchronous content in learning management systems or other accessible platforms. These adaptations reflect a broader trend toward meeting library users where they are – both literally and pedagogically – and aligning services to the structure of PA education. For example, librarians have adapted collection development by attuning to everyday signals from their PA communities; monitoring interlibrary loan requests, reviewing syllabi, or picking up on research topics and themes in faculty conversations. Without formal guides or centralized input, librarians anticipate needs in real time through benchmarking, informal feedback, and maintaining a presence in the academic space, underscoring the value of being immersed in the environment they support.

The challenges of supporting PA programs have highlighted the need for institutional and professional support for librarians themselves. As discussed, interviewees emphasized the impact of the learning curve in librarians’ liaison workload. Through the conversations, they shared the importance of workload planning, targeted training, and peer networks, suggesting that professional organizations supporting health sciences librarians have opportunities to build on targeted professional development and networking. However, academic libraries must also be intentional about staff support, particularly workload distribution and professional development, ensuring that time, space, and resources are allocated to equip librarians whether they are launching a new academic program, are new to PA librarianship, or are new to the profession in general.

## LIMITATIONS

Online surveys have many advantages, such as easy administration, quick distribution across platforms, and simplified data analysis. However, they can carry significant drawbacks; they are susceptible to selection bias due to a convenience sample, which may reflect non-response bias and does not represent the broader population accurately. Consequently, the findings may not truly reflect the diverse perspectives or experiences of the larger community.

Semi-structured interviews offer rich qualitative insights into interviewees’ thoughts and perceptions. They are also susceptible to selection bias as well as researcher bias, and social desirability bias from the interviewees.

## Data Availability

Data associated with the semi-structured interviews in this article cannot be made publicly available because they contain personally identifiable information. Access to the survey data can be requested from the corresponding authors and may be subject to IRB restrictions.

## SUPPLEMENTAL FILES

**Appendix A:** Survey Questions

**Appendix B:** Interviewees’ Type of Institution and Length of Time Since Accreditation According to ARC-PA

**Appendix C:** Initial Interview Questions
