# Supplementary material for: Navigating unique challenges: librarian perceptions in supporting physician associate (assistant) programs
Source: J Med Libr Assoc. 2026 Feb 17;114(1):21–30. doi: 10.5195/jmla.2026.2211 (PMC12947935; doi:10.5195/jmla.2026.2211)
Supplement: Supplementary file 1 — Appendix A: Survey Questions [file jmla-114-1-21-s01.docx]

Survey Questions

1. How long has your institution had or will have a PA program?
   1. 0-5 years
   2. 5 or more years
   3. Program is in the accreditation process and will be taking students in the next few years
2. Which best describes the type of institution you work for
   1. University/College with a dedicated health sciences library or medical school
   2. Research university with neither a medical school nor a dedicated health sciences library
   3. University/College with graduate degrees
   4. Other please explain:_____________________
3. Which of the following types of instruction or orientation sessions do you have or plan to have for the PA program? (check all that apply)
   1. General orientation sessions
   2. Class based instruction
   3. Workshops
   4. Library instruction modules or tutorials
   5. Other please explain:___________________
4. If you do library instruction with the PA students, which of the following types of classes is it associated with?
   1. Didactic
   2. Clinical
   3. Both
5. If you do library instruction with the PA students, which of the following topics are included in these sessions? (check all that apply)
   1. General search skills
   2. Specific clinical health science tools
   3. Developing search strategies
   4. Evidence Based Practice
6. Aside from the PA program, which of the following medical/health sciences programs does your institution have? (check all that apply)
   1. Biomedical science or biosciences
   2. Dental
   3. Exercise science/athletic training/kinesiology
   4. Medicine
   5. Nursing
   6. Osteopathic medicine
   7. Pharmacy
   8. Physical or Occupational Therapy
   9. Public Health
   10. Other please explain:___________________
7. What products do you promote to the PA students and faculty? (Check all that apply)
   1. AccessMedicine
   2. Uptodate
   3. Dynamed
   4. Lexicomp
   5. StatRef
   6. PubMed (or Medline)
   7. CINAHL
   8. Clinical Key
   9. Visual DX
   10. Other please explain:__________________
8. Does your PA program have a research component to it?
   1. Yes
   2. No
9. If the answer to 8 is yes, please explain.
   1. ______________________________________
10. Are you willing to participate in a 30-40 minute interview about your experiences with PA students/faculty?
    1. Yes, please contact me for further information
    2. No.
