# Supplementary material for: Navigating unique challenges: librarian perceptions in supporting physician associate (assistant) programs
Source: J Med Libr Assoc. 2026 Feb 17;114(1):21–30. doi: 10.5195/jmla.2026.2211 (PMC12947935; doi:10.5195/jmla.2026.2211)
Supplement: Supplementary file 2 — Appendix B: Interviewees’ Type of Institution and Length of Time Since Accreditation According to ARC-PA [file jmla-114-1-21-s02.docx]

**Appendix B.** Interviewees’ type of institution and length of time since accreditation according to ARC-PA. Type of Institution: A=University/College with a dedicated health sciences library or medical school. B=Research university with neither a medical school nor a dedicated health sciences library, and C=University/College with graduate degrees. PA Program Length of Time since Accreditation (at time of survey): A=0-5, B=5-10, and C=10 or more years.

| Interviewee | Type of Institution | PA Program Length of Time since Accreditation |
| --- | --- | --- |
| 1 | C | A |
| 2 | B | B |
| 3 | A | C |
| 4 | A | C |
| 5 | C | A |
| 6 | C | C |
| 7 | C | A |
| 8 | C | C |
| 9 | A | C |
| 10 | B | A |
| 11 | A | C |
| 12 | B | B |
| 13 | A | A |
| 14 | A | B |
| 15 | B | A |
