# Supplementary material for: Navigating unique challenges: librarian perceptions in supporting physician associate (assistant) programs
Source: J Med Libr Assoc. 2026 Feb 17;114(1):21–30. doi: 10.5195/jmla.2026.2211 (PMC12947935; doi:10.5195/jmla.2026.2211)
Supplement: Supplementary file 3 — Appendix C: Initial Interview Questions [file jmla-114-1-21-s03.docx]

Follow the survey and turn closed questions into open-ended questions

Warm up question: Tell me about your job.

Probe/Follow-Up: For how long have you worked at your present institution?

Probe/Follow-Up: For how long have you been in this role?

(Ask question 1a or 1b depending on their survey response of having a PA program or in development)

1a. In the survey you indicated that your institution has had a PA program for____ years. Please share more about the program and your (individual/library) involvement.

1b. In the survey you indicated that your institution is developing a PA program. Where is the institution in this process? What has been your (individual/library) involvement?

2. In the survey you mentioned your institution also has _____(list of medical/health science programs)____. How is the PA program tied to other allied health programs at your institution? What similarities do they have? What differences?

3. What library resources are used most by PA faculty and students?

Probe/Follow-Up: What resources do you wish they used instead?

Probe/Follow-Up: Books? Ebooks? Journals? 3D models? Space? Tools?

Probe/Follow-Up: Resources/services for studying for boards

4. How are the resources funded? How does the library determine what is needed? (sit on committees, general faculty/student feedback, guess?)

Probe/Follow-Up: are certain e- resources or physical resources available only for PA students?

5.Please tell us about your experience collaborating with PA faculty, staff and students.

Probe/Follow-Up: Do you collect faculty/student feedback? Can you share about this?

Probe/Follow-Up: Are you involved in any way with the PA program’s curriculum planning?

6.In the survey you indicated that you facilitate learning opportunities specifically for the PA program. Please tell us about these offerings and interactions?

Probe/Follow-Up: How much control do you have over these offerings and interactions?

Probe/Follow-Up: Is library instruction scaffolded throughout the program? One-shots? Embedded librarianship?

7. What are some barriers (institutional, cultural, programmatic, etc.) for supporting the PA program?

Probe/Follow-Up: If there was one thing you could change about your work with the PA program, what would it be?

8. What are the chief facilitators (institutional, cultural, programmatic, etc.) for supporting the PA program?

9. Is there anything else you would like to tell us? Or do you have any questions for us?
